# Refractory Gastroesophageal Reflux Disease: A Management Update

**DOI:** 10.3389/fmed.2021.765061

**Published:** 2021-11-01

**Authors:** Francesco Rettura, Francesco Bronzini, Michele Campigotto, Christian Lambiase, Andrea Pancetti, Ginevra Berti, Santino Marchi, Nicola de Bortoli, Frank Zerbib, Edoardo Savarino, Massimo Bellini

**Affiliations:** ^1^Division of Gastroenterology, Department of Translational Research and New Technologies in Medicine and Surgery, University of Pisa, Pisa, Italy; ^2^Department of Medicine, Surgery and Health Sciences, University of Trieste, Trieste, Italy; ^3^CHU de Bordeaux, Centre Medico-Chirurgical Magellan, Hôpital Haut-Lévêque, Gastroenterology Department, Université de Bordeaux, INSERM CIC 1401, Bordeaux, France; ^4^Division of Gastroenterology, Department of Surgery, Oncology and Gastroenterology, University of Padua, Padua, Italy

**Keywords:** gastroesophageal reflux disease (GERD), refractory GERD (rGERD), 24-h multichannel intraluminal impedance-pH (MII-pH), proton pump inhibitors (PPIs), laparoscopic antireflux surgery (LARS)

## Abstract

Gastroesophageal reflux disease (GERD) is one of the most frequent gastrointestinal disorders. Proton pump inhibitors (PPIs) are effective in healing lesions and improving symptoms in most cases, although up to 40% of GERD patients do not respond adequately to PPI therapy. Refractory GERD (rGERD) is one of the most challenging problems, given its impact on the quality of life and consumption of health care resources. The definition of rGERD is a controversial topic as it has not been unequivocally established. Indeed, some patients unresponsive to PPIs who experience symptoms potentially related to GERD may not have GERD; in this case the definition could be replaced with “reflux-like PPI-refractory symptoms.” Patients with persistent reflux-like symptoms should undergo a diagnostic workup aimed at finding objective evidence of GERD through endoscopic and pH-impedance investigations. The management strategies regarding rGERD, apart from a careful check of patient's compliance with PPIs, a possible change in the timing of their administration and the choice of a PPI with a different metabolic pathway, include other pharmacologic treatments. These include histamine-2 receptor antagonists (H2RAs), alginates, antacids and mucosal protective agents, potassium competitive acid blockers (PCABs), prokinetics, gamma aminobutyric acid-B (GABA-B) receptor agonists and metabotropic glutamate receptor-5 (mGluR5) antagonists, and pain modulators. If there is no benefit from medical therapy, but there is objective evidence of GERD, invasive antireflux options should be evaluated after having carefully explained the risks and benefits to the patient. The most widely performed invasive antireflux option remains laparoscopic antireflux surgery (LARS), even if other, less invasive, interventions have been suggested in the last few decades, including endoscopic transoral incisionless fundoplication (TIF), magnetic sphincter augmentation (LINX) or radiofrequency therapy (Stretta). Due to the different mechanisms underlying rGERD, the most effective strategy can vary, and it should be tailored to each patient. The aim of this paper is to review the different management options available to successfully deal with rGERD.

## Introduction

Gastroesophageal reflux disease (GERD) is one of the most frequent gastrointestinal diseases (1). It is defined on the basis of both esophageal and extra-esophageal symptoms, and/or lesions resulting from the reflux of gastric contents into the esophagus. GERD symptoms can be typical, such as heartburn and regurgitation, and atypical, such as chest pain, chronic cough, laryngeal burn, globus, and hoarseness. Therapy is commonly based on proton pump inhibitors (PPIs) and alginates as an add-on therapy. PPIs are effective in healing lesions and improving symptoms in most cases (2). However, there is a significant proportion of patients, ranging from 10 to 40%, whose symptoms do not adequately respond to PPI therapy (3–6). This condition, commonly known as “refractory GERD” (rGERD), represents a major health problem, given its impact on quality of life and consumption of health care resources (7). The definition of rGERD is controversial as it has never been clearly established (8). The most commonly used definition is: symptoms (retrosternal heartburn and/or regurgitation) present at least 3 times per week not responding to a double dose of PPIs for 8–12 weeks (4, 7–10). It must be emphasized that this definition is only clinical, and it does not take into account the need to have objective evidence of GERD based on endoscopic findings and pH-impedance monitoring. Indeed, many patients who experience symptoms potentially related to GERD and not responding to PPI are not really affected by GERD (7, 9). In this case the definition could be changed to “reflux-like PPI-refractory symptoms.” The latest ESNM/ANMS consensus paper (11), in accordance with recent recommendations (12–14), defined “refractory GERD symptoms” as the persistence of symptoms on therapy, in patients with prior objective evidence of GERD (erosive esophagitis, peptic stricture, long segment Barrett's esophagus, or abnormal esophageal acid exposure on reflux monitoring performed off therapy) and rGERD as “persistence of GERD symptoms with objective evidence of GERD (through endoscopic and pH-impedance findings) despite optimized PPI therapy over at least 8 weeks.” The complex pathogenetic mechanisms underlying rGERD represent a major challenge in gastroenterological clinical practice and need to be further investigated in order to guide effective therapeutic interventions (15). The present paper was aimed at reviewing the treatment of rGERD in light of the most recent research.

**Clinical tip: rGERD is referred to the persistence of GERD symptoms with objective evidence of GERD despite optimized PPI therapy over at least 8 weeks**.

## Diagnostic Workup

Patients with persistent reflux symptoms should undergo a diagnostic pathway aimed at confirming the diagnosis of GERD, evaluating potential comorbidities (including obesity, cardiological and respiratory diseases, psychological/psychiatric disorders) (16), presence of gut-brain axis disorders (17–20) and the use of concomitant medications (21) (Table 1).

**Table 1:** Drugs commonly associated with esophageal damage and/or onset of reflux-like symptoms.

Statins (i.e., Simvastatin, Rosuvastatin)
Angiotensin converting enzyme-inhibitors (i.e., Ramipril)
Selective serotonin reuptake inhibitors (i.e., Fluoxetine)
Calcium channel blockers
Antiplatelets (i.e., Clopidogrel)
Antibiotics (i.e., Clindamycin or Doxycycline)
Ferrous sulfate
NSAIDs (i.e., Aspirin, Ibuprofen, Naproxen)
Theophylline
Nitrates
Phosphodiesterase 5 inhibitors (i.e., Sildenafil, Tadalafil)
Diphosphonates

*NSAIDs, non-steroidal anti-inflammatory drugs*.

An esophagogastroduodenoscopy (EGD), with mucosal biopsies when necessary, should be suggested in order to identify the presence of erosive esophagitis (and its complications) and/or to rule out other causes of esophageal damage, like eosinophilic esophagitis (22, 23). If negative, other tests should be performed (24). High-resolution esophageal manometry (HREM) should be carried out to detect non-reflux esophageal disorders (10, 25), such as esophageal motility disorders, which may have similar complaints of patients with GERD (26–29). If symptoms are suspected of being due to delayed gastric emptying, an assessment of gastric emptying (scintigraphy or breath test) is recommended (30, 31) (Table 2).

**Table 2:** Possible causes of persistent GERD-like symptoms and corresponding diagnostic tests.

**Possible causes of persistent GERD-like symptoms**	**Diagnostic test**
Eosinophilic esophagitis	EGD
Zollinger-Ellison syndrome	
Pill-induced esophagitis	
Skin disease with esophageal involvement	
Infectious esophagitis	
Esophageal cancer	
Radiation-induced esophagitis	
Caustic agent ingestion	
Esophageal motility disorders (Achalasia, hypotensive LES, reduced esophageal contractility)	HREM
Supragastric belching	
Rumination syndrome	
Reflux hypersensitivity	MII-pH
Functional heartburn	
Delayed gastric emptying	Gastric emptying scintigraphy or Breath test

*EGD, esophagogastroduodenoscopy; GERD, gastroesophageal reflux disease; HREM, high resolution esophageal manometry; MII-pH, 24-h multichannel intraluminal impedance-pH; LES, lower esophageal sphincter*.

HREM is also mandatory in order to correctly perform a 24-h multichannel intraluminal impedance-pH (MII-pH) monitoring, the current gold standard test to verify the presence of reflux and its association with symptoms (13, 32, 33). MII-pH monitoring assesses the chemical composition of reflux (acidic, weakly acidic, or non-acidic based on pH), its physical composition (liquid, gaseous, or mixed), and its migration to the distal esophagus (34). This technique, in combination with the analysis of the relationship between symptoms and reflux events [symptom index (SI) or symptom association probability (SAP)], determines the association of reflux events with GERD symptoms (7, 15, 35, 36). The GERD Consensus Group considers an acid exposure time (AET) to be normal when <4% and pathological when >6%. For borderline values (between 4 and 6%) the Group suggests considering other metrics, e.g., the number of reflux episodes (acidic, weakly acidic or weakly alkaline), where more than 80 reflux episodes per 24 h are definitely considered abnormal. However, it has also been reported that the total number of reflux episodes alone is not sufficient to confirm the diagnosis of GERD and it should be considered only as an exploratory tool (13, 37).

MII-pH monitoring leads to a stratification of patients into ongoing acid reflux, ongoing symptomatic non-acid reflux (when AET is normal but the patient reports symptoms corresponding to non-acid or weakly acid reflux events) and non-reflux (when AET is normal and reported symptoms are independent of reflux events) (25, 38–41). The test carried out “off treatment,” i.e., after having suspended any acid-suppressive therapy for at least 20 days, confirms the diagnosis of GERD (NERD type), discriminating between GERD functional esophageal disorders like reflux hypersensitivity (RH) and functional heartburn (FH) (42). The latest ESNM/ANMS consensus paper emphasizes the importance of considering additional impedance parameters, such as SAP, post-reflux swallow-induced peristaltic wave index, and mean nocturnal baseline impedance, in order to help identify the patients with rGERD presenting with an inconclusive “off treatment” test (11). MII-pH performed “on – treatment” (during PPI intake) aims to find the causes of a treatment failure (25, 43) and is reserved for patients previously diagnosed with GERD (e.g., affected with Los Angeles Grade C or D esophagitis, peptic stricture, Barrett esophagus, or pathological acid exposure off PPI on esophageal pH monitoring) not responsive to an optimized PPI trial. MII-pH monitoring is essential to assess not only a PPI-refractory acid exposure but also an excessive burden of weakly-acidic contents (37). Identifying the rGERD patient phenotype through MII-pH monitoring is crucial to enable proper management on the basis of the pathophysiological mechanisms (25, 44).

**Clinical tip: 24 h MII-pH monitoring “on - treatment” is the gold standard in the diagnostic pathway for rGERD, it being crucial in confirming the presence of reflux and its association with symptoms**.

## Potential Causes of rGERD

### PPI Compliance

In rGERD patients it is necessary to evaluate compliance with the therapy, both in terms of dose and timing of drug intake (45). Dose and timing are key factors in obtaining an adequate response. PPI should be taken when fasting, at least 30 min before a meal, preferably in the morning before breakfast or, in the case of a second dose in the evening before dinner. This is to achieve maximum gastric acid suppression, blocking the proton pumps before food activates them (3). Up to 54% of patients take PPI incorrectly (46) and several studies have shown a poor adherence to PPI therapy, with two reports finding that only 53.8 and 67.7% of patients adhered correctly kept to the prescription for more than 80% of the expected time (47, 48). In addition, many patients stop treatment when their symptoms improve. This was shown by a large population survey in which only 55% of patients took PPI once a day for 4 weeks as prescribed, with 37% taking the drug for 12 days or less (49). Moreover, a study involving 100 patients with persistent GERD-related symptoms found that only 8% of them took the PPI therapy 30–60 min before meals as prescribed (47). Factors related to poor adherence are mainly the presence of mild symptoms, the onset of side effects, poor information, and poor knowledge on the part of the physician regarding the more detailed pharmacological characteristics of PPIs (50).

### PPI Metabolism

The pharmacological activity of PPIs differs among patients. A potential contributing factor in this regard is the genotypic variability of cytochrome P450 (CYP) (51). PPIs are metabolized by the liver enzyme complex of CYP, mainly by CYP2C19, and to a lesser extent by CYP3A4. There are three polymorphic variants of CYP2C19 corresponding to 3 different phenotypes: extended or rapid metabolizer (RM) (homozygous, with two mutated alleles), intermediate metabolizer (IM) (heterozygous, one mutated allele and one wildtype) and slow metabolizer (SM) (two wild-type alleles). The rapid metabolizing variant is present in about 60–70% of Caucasians and 30–40% of Asians (52). In a recent meta-analysis, it was found that the response rate to PPIs in RM is lower (52.2%) than in IM (56.7%) and in SM (61.3%) (53). The role of CYP2C19 activity is still unclear. There is only one study in which a discrepancy between a single and double dose of Pantoprazole in RMs is observed, and this could represent a starting point for future studies (54). Up to date genotyping of CYP2C19 is possible by polymeric chain reaction on a peripheral blood sample, but it is not yet widely available for clinical practice (55).

### Different PPI Agents

Evidence of the superiority of a PPI agent in comparison with others for rGERD is scarce, but some data suggest that different types of PPI activity can vary widely in terms of acid suppression (56), so some patients could exhibit significant variability in their clinical response to different molecules. Hence, when considering large cohorts of patients there is no evidence of superiority of one PPI over another, whereas in the individual patient it is sometimes useful to switch to a different PPI to obtain better symptom control (57).

### Persistent Weakly Acid or Non-acid Reflux

Stimuli other than strong acidity can also be responsible for reflux symptoms, i.e., weakly acid reflux (WAR) or weakly alkaline reflux (WalkR). WalkR has a pH > 7 and WAR has a pH between 4 and 7. It has been observed that between 30 and 40% of patients with rGERD have symptoms related to events of WAR and WalkR (7–10, 15, 58–60). It should not be forgotten that in patients taking PPI, reflux episodes are more frequently of weakly acidic and weakly alkaline nature than acidic as a direct consequence of acid suppression (35). The most widely accepted hypothesis is that distension of the esophagus due to reflux volume and esophageal hypersensitivity to reflux are the conditions that trigger the symptoms (61). It has also been hypothesized that lower pH values of WAR could be a determinant factor in provoking heartburn (39, 62, 63). During a weakly acidic reflux event, pepsin can migrate into the esophagus. This has a proteolytic effect, and although its maximum activity is between pH 1.9–3.6, it displays partial activity even above pH 6 (61). Moreover, it has also been observed that up to pH 6.5, mucosal repair may be impaired (61) and previous exposure of the mucosa to acidic pH may result in the development of hyperalgesia to both mechanical and chemical stimulation (64).

### Duodenogastroesophageal or Bile Reflux

Many physicians confuse WAR and bile reflux. The latter is weakly alkaline and can be found especially in patients who have undergone gastrectomy. Bilirubin and bile acids can damage the esophageal mucosa, causing apoptosis, appearance of dilated intercellular spaces (DIS), and increased mucosal permeability (7, 53, 65–67). Data on the role of bile reflux in rGERD are controversial. In a study by de Bortoli et al. bile reflux was shown to play a role in the origin of reflux symptoms not responding to acid suppression (68). However, Gasiorowka et al. found no difference in the degree of duodenogastroesophageal reflux and acid exposure during treatment between patients who failed to respond and those who achieved complete symptom resolution after a PPI trial (69).

## Pathophysiology of rGERD

### Antireflux Barrier

The weakening of the physiological antireflux barrier seems to play a role in promoting rGERD (3, 9). The antireflux barrier is a high-pressure area consisting of the lower esophageal sphincter (LES) attached to the crural diaphragm through the esophageal ligament, and it prevents gastroesophageal reflux. A reduction in the mucosal barrier's integrity is mainly determined by reduced LES resting pressure, especially in the presence of hiatal hernia, and transient relaxation of LES (TLESR), mainly in the presence of an intact hiatus; this may lead to an increase in volume and exposure time of the refluxed material (3, 12, 21, 70). Zerbib et al. (11) reiterated the potential contribution of obesity (especially central obesity) in promoting rGERD through increasing gastric pressure, leading to higher TLESR events. Further studies are underway to clarify the importance of a weakening of the antireflux barrier in the pathogenesis of rGERD (24, 71–73).

### Esophageal Clearance

It has been observed that a delayed clearance of acid and bolus from the esophagus may be associated with a persistence of pathologic reflux, particularly following conditions such as altered esophageal peristalsis, hiatal hernia, and alterations in salivation (74–78). One study has also observed that PPI- refractory patients have lower basal levels of esophageal clearance, which may worsen the effect of reflux (79).

### Delayed Gastric Emptying

The association between delayed gastric emptying and rGERD remains unclear (80). It has been hypothesized that delayed gastric emptying, or gastroparesis, may contribute to rGERD through increased gastric distension and consequent TLESR events, which promote reflux of gastric content into the esophagus (81). In one study the use of a prokinetic agent, accelerating gastric emptying prior to pH-impedance, and manometry tests significantly reduced AET and clearance (82).

### Esophageal Hypersensitivity and Hypervigilance

There is growing consensus that esophageal hypersensitivity plays an important role in rGERD, particularly in the presence of NAR related symptoms (83, 84). It is defined as the increased perception of different kinds of stimuli, including acid, temperature, mechanical distension, and electrical stimulation (70). The pathophysiologic mechanism seems to involve central and peripheral sensitization through the presence of DIS and exposure of subepithelial nerves to acid. Chronic psychological stress can induce disruption of the intestinal epithelial tight junction proteins, leading to increased intestinal epithelial permeability and associated visceral hyperalgesia (7, 65–67, 84). Recently, the new concept of esophageal hypervigilance, defined as the cognitive-affective process that results from over-awareness of the discomfort, was introduced by Keefer et al. (85) as a possible factor involved in rGERD. The analysis of 70 patients with PPI refractory symptoms studied with pH-impedance and psychometric tests showed that the hypervigilance was shared by all phenotypes, regardless of the patient's phenotype in terms of positive or negative correlation of symptoms and normal or abnormal acid exposure (85).

## Therapy of rGERD

**Figure 1:**
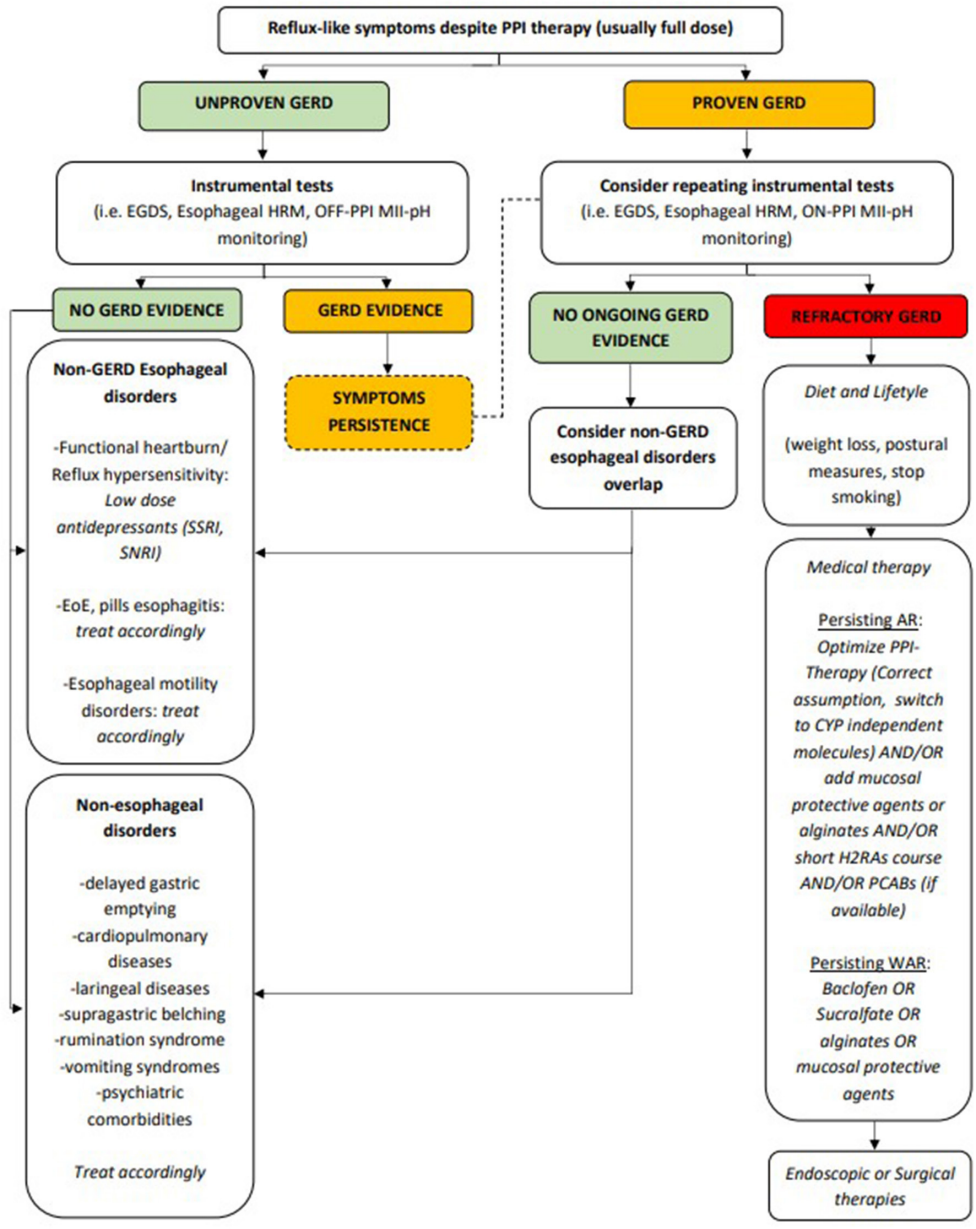
Proposed management of rGERD symptoms. AR, acid reflux; CYP, cytochrome P450; EGDS, esophagogastroduodenoscopy; EoE, eosinophilic esophagitis; GERD, gastroesophageal reflux disease; H2RAs, histamine receptor 2 antagonists; HRM, high resolution manometry; MII-pH, multichannel intraluminal impedance-pH; PCABs, potassium-competitive acid blockers; PPI, proton pump inhibitor; SNRI, serotonin and norepinephrine reuptake inhibitor; SSRI, selective serotonin reuptake inhibitor; WAR, weakly acid reflux.

As previously described, several different mechanisms can lead to rGERD and therapy should target specific pathophysiological events. However, in clinical practice, drug use is often aimed at controlling refractory symptoms, especially refractory heartburn or refractory epigastric pain (Figure 1).

### Diet and Lifestyle

In patients with GERD the first life-style change should consider modification in body weight and elevation of the head of the bed (86–88). Weight loss of at least 10% is recommended in all patients with GERD in order to boost the effect of PPI on symptom relief and to reduce chronic medication use (88). Indeed, potentially effective dietary measures in improving GERD symptoms are those aimed at weight loss, although with a moderate level of evidence (89, 90), and those improving esophageal motility, such as supplementation of dietary fibers. A recent study shows that Psyllium 15 g/day can significantly increase LES minimal resting pressure in NERD patients, leading to a reduction in the number of acid and weakly acid reflux events and in the frequency of pyrosis (91). Recently, a study based on MII-pH post-prandial analysis by Martinucci et al. showed that a meal based on vegetable proteins was associated with a lower number of refluxes, particularly acid refluxes, and with a reduced number of symptoms during the first postprandial hour, compared to one with a prevailing component of animal proteins (92). Also, regarding avoidance of alcohol, peppermint, coffee or fatty food, evidence is scarce and this suggestion, despite being very frequently proposed in clinical practice, cannot be strictly recommended (90, 93, 94). A clinical trial showed that fermentable oligo-, di-, mono- saccharides and polyols (FODMAPs), particularly fructans, increased the number of TLESRs in healthy patients (95), and in proven rGERD patients a low- FODMAP diet was shown not to significantly decrease reflux symptoms when compared to usual dietary advice (96). In conclusion, no clear scientific evidence regarding a positive role of dietary suggestions in rGERD exist to date, even if also in these patients they are frequently recommended because they are low cost and can lead to positive effects on individual health. On the contrary, the association between tobacco smoking and GERD is well-documented by a recent meta-analysis (97). Reinforcing avoidance of tobacco consumption in refractory patients could thus be a cost/effective measure.

**Clinical tip: weight loss, postural measures and smoking cessation are cost-effective also in rGERD patients**.

### PPI Therapy

As previously described, PPIs are the standard therapy for GERD. When symptoms persist, revealing a condition of rGERD, many different possible therapeutic options are available despite their efficacy often being weak (98).

#### Reinforcing Correct Drug Intake

Low adherence regarding PPI intake is described in up to 47.5% of cases, as mentioned above (99). Stressing the instructions for correct drug intake (i.e., taking it with an empty stomach, followed by a meal within 30 min) is however essential (54), in order to obtain maximum cost-effectiveness in acid gastric suppression.

#### Doubling PPI-Dose and High PPI-Doses

According to the above-mentioned definition, patients with rGERD should already have experienced a double-dose PPI treatment. A twice-daily PPI dose is not recommended by European and North American Drug Authorities. It can however lead to adequate symptom control after 6–8 weeks in 20–30% of patients who have been on a standard daily dose, when persistent acid exposure is proved by MII-pH monitoring (100). Whether clinical remission is achieved or not, titration should be proposed after 2–3 months, in order to avoid possible long-term PPI side effects (25, 101). Finally, only a recent controlled trial recorded a strong pH-lowering (MII-pH monitoring) when Esomeprazole 20 mg q.i.d. was administered to Helicobacter Pylori (HP) negative patients (102). The authors concluded that this might be one of the rescue regimens for patients who are refractory to PPI treatment, irrespective of CYP2C19 genotype. However, caution is mandatory until more robust evidence is available.

#### Changing PPI Formulation

In order to overcome compliance problems, new drug formulations such as Dexlansoprazole modified-release (MR) have recently been developed. This MR formulation allows for a once-daily dosage regardless of time of day or food consumption. However, the drug has been mainly tested in patients with erosive esophagitis or nocturnal GERD symptoms, rather than in patients with true rGERD (103). It is also more expensive than standard PPI formulations and it is not widely available in all European markets. No other PPI MR formulations have been studied to date.

#### Splitting PPI-Dose

This can be suggested when there are persistent nocturnal symptoms, possibly associated with NAB. Papers describing the effects of splitting PPI doses on gastric acidity and especially on NAB, differ according to the different PPI molecules. A pH increase has been described when Omeprazole 40 mg once a day is split into two doses (i.e., before breakfast and dinner) (104), but not when splitting Lansoprazole 30 mg or Pantoprazole 40 mg (105). This is despite the same efficacy as Omeprazole being achieved when these molecules are given once a day. A more recent study in healthy volunteers showed that Esomeprazole can lead to a better acid secretion control in the initial 7-day period of the treatment when the daily dose (40 mg) is split into a morning and an evening dose (106).

#### Changing PPI-Molecule or PPI-Brand

Although evidence is quite limited for a PPI agent being superior to the others for rGERD, some data suggest that relative potencies of different PPIs vary widely (107, 108). Therefore, as previously mentioned, patients can exhibit significant variability in their clinical response to different molecules (56). However, a recent meta-analysis found no differences in the effectiveness of acid suppression when comparing equivalent doses of different types of PPIs, indicating that these can be used interchangeably (56). Moreover, the 2005 Canadian Consensus Conference (109) and the statements of the World Health Organization Collaborating Centre for Drug Statistics Methodology have suggested that PPIs are more similar than different (110). Pending more robust data, an attempt to change the molecule can be made, although it is difficult to quantify the effectiveness of this measure. In these cases, drug doses should be titrated according to the described relative power. Choice of a CYP-independent PPI requires further consideration and can be seen as a different strategy, as described in the next paragraph. Finally, there are very few reports regarding the possible efficacy of simply changing the PPI brand within the same molecule (111). In our opinion, in this case, when patients report improvement of symptoms, a placebo effect and/or psychological component could play an important role, especially when there are other functional disorders and/or psychiatric comorbidities involved.

#### Switch to a CYP-Independent PPI

Most PPIs, except Esomeprazole and Rabeprazole and where available Ilaprazole and Azelaprazole, undergo hepatic metabolism through the CYP enzymatic complex, mainly through CYP2C19 (112) and CYP3A4 isoenzymes (113). As previously described, patients can be classified into RM, IM and SM. There is a higher prevalence of RM patients among Caucasians (60–70% of the general population) and Asian populations (82). Genetic testing evaluating the CYP2C19 genotype is quite expensive and seldom available thus making a PPI shift to CYP independent molecules the commonest clinical approach (55). Taken together, both doubling PPI dose (among common CYP- dependent molecules) and switching to a CYP-independent molecule account for a 25% improvement overall in satisfactory clinical response and, despite the greatest potential gain for the second strategy, there is no solid evidence to date for either one or the other (100). The most important aspect to consider is that the relative response of different symptoms to acid suppression correlates with their association with acid reflux (114, 115) and up to 80% of patients with persistent symptoms, despite optimized PPI therapy, probably have non-acid reflux. In these cases other therapeutic approaches should be considered.

**Clinical tip: PPI are more effective when taken with an empty stomach and 30 min before breakfast. It could also be useful switching to CYP independent PPI or splitting PPI into two doses if nocturnal symptoms are present**.

### Histamine-2 Receptor Antagonists

An eventual effective therapeutic strategy can involve the addition of a Histamine-2 receptor antagonist (H2RA) at bedtime to control nocturnal symptoms (14). This is because histamine is an important driver of nocturnal acid secretion and may explain the scarce responses to PPI bedtime administration (99). H2RAs could also modulate esophageal sensitivity to acid in patients with GERD or FH (116). Some studies have shown that the addition of a nighttime H2RA dose to twice- daily PPI decreases nocturnal acid breakthrough (NAB) from 64 to 17% (55, 117–119). However, other research has not clearly detected a correlation between NAB and symptoms and has not shown a significant reduction in AET and SI (117). Moreover, tachyphylaxis phenomena can develop in <10 days after starting H2RA therapy (118), leading to drug discontinuation in up to 13% of patients (119). Hence, H2RA would be better taken on demand or intermittently (9), regardless of whether it is an add-on therapy to a double PPI therapy or a substitute for the second PPI dose (25). It is worth noting that administration of H2RAs together with PPIs is considered to be safe (120).

**Clinical tip: addition of a H2RAs at bedtime can be considered to better control nocturnal symptoms**.

### Alginates, Antacids, and Mucosal Protective Agents

This is a wide class of drugs with different mechanisms of action, effective in controlling residual symptoms generally without serious side effects. Solutions containing sodium alginate precipitate into a viscous gel that may create a physical barrier to the so-called “acid pocket,” which may not be completely eliminated by PPIs (79). These drugs decrease the severity and frequency of heartburn when used postprandially as add-on therapy to PPIs in patients with GERD (121). It is noteworthy to mention that alginates available on the U.S. and European markets are different, with the latter containing higher alginate concentrations and leading possibly to a higher efficacy (35). Despite some studies reporting prompt efficacy in controlling both postprandial and nocturnal symptoms in patients with rGERD (121), none of these objectively assessed presence and type of reflux with MII- pH monitoring. Given their lack of significant adverse events, they can however be used as an add-on to PPIs according to local availability and the physician's and patient's preference. Sucralfate is a mucosal protective agent that acts by blocking the diffusion of gastric acid and pepsin through the esophageal mucosa and stimulates mucosal growth factors, thus promoting the formation of mucus and bicarbonate (122). It is a safe drug, with a good efficacy in controlling GERD symptoms and a potential effectiveness in improving mucosal healing of erosive mild grade esophagitis. Despite the lack of data regarding its use in patients with rGERD (123), sucralfate may be a safe option for symptom management in pregnant women (124) and, as a complementary therapy in patients with drug-induced esophagitis (122). Due to the ability of binding bile salts, it could be of help also in WAR and/or WalkR. A new class of disposable medical agents, made up of a bioadhesive formulation of hyaluronic acid and chondroitin sulfate, was recently shown to improve symptoms and quality of life as an add-on therapy to PPI when compared to PPI alone in patients with NERD (125, 126). Despite their use in patients with rGERD not having been evaluated yet, they could be of help in some patients because they target an additional pathophysiological mechanism.

**Clinical tip: adding alginates, antacids or mucosal protective agents can be effective in controlling residual symptoms, generally without serious side effects**.

### Potassium-Competitive Acid Blockers

Potassium-competitive acid blockers (PCABs) competitively inhibit proton pumps (hydrogen- potassium ATPases) and have been approved in Japan since 2015 for the treatment of peptic ulcer and reflux esophagitis and eradication of HP infection (127, 128). Given their clinical efficacy, experts suggest that they could also be more useful than PPIs as an initial empirical test before performing instrumental diagnostic tests to exclude acid-mediated reflux disease (129). Vonoprazan, which is not yet available on the European market, has shown a greater ability in suppressing acid secretion through blocking both inactive and active proton pumps, with a greater duration of action compared to PPIs (130–132). Its elimination is independent of CYP2C19 and this may contribute to explaining its stronger effect (133). Some retrospective studies have demonstrated symptomatic improvement in patients with rGERD (134) and efficacy in the treatment of persistent symptomatic erosive esophagitis when drug intake is continued for at least 8 weeks. Furthermore, a very recent small (30 patients) uncontrolled study showed the long-term efficacy with 1 year of Vonoprazan in rGERD (135). However, a study in rGERD patients complaining of dyspepsia was not able to detect any significant effect of Vonoprazan (136) and the drug failed to normalize AET in patients with absent esophageal contractility (i.e., patients with systemic scleroderma) (137).

**Clinical tip: whenever available, vonoprazan is more effective than PPIs in acid suppression**.

### Prokinetics

Prokinetic agents, approved for the treatment of patients with gastroparesis (138), have also been suggested as an add-on therapy in some patients with rGERD, since delayed gastric emptying can lead to symptom persistence. These drugs could act not only by improving gastric emptying, but also by increasing LES pressure and improving esophageal clearance. According to the different molecules, they can act by binding to different receptors, including 5-hydroxytryptamine (5-HT) 4, dopamine 2 (D2), motilin and ghrelin receptors (80). However, a recent meta-analysis found that the addition of a prokinetic to a PPI does not provide clear benefits in symptom control, but it improves quality of life (139). Hence, in rGERD patients with normal gastric emptying, adding these agents for a short time-period deserves to be carefully evaluated in terms of risk-benefit balance.

#### Anti Dopamine 2 Receptor Agents

Metoclopramide, an anti-dopaminergic drug, has been studied in association with H2RAs, but it did not significantly improve symptoms over H2RAs alone (140). Studies to assess its efficacy as an add- on therapy to PPIs in rGERD patients are lacking. However, its long-term use (i.e., more than 12 weeks) could be limited by the onset of side effects like insomnia, agitation and tardive dyskinesia. Also, Domperidone has been shown to be effective in increasing LES pressure (141), but a recent study indicated that it did not improve reflux symptoms when added to PPI in rGERD (142), thus limiting its use within this context.

#### 5-HT Receptor Active Drugs

Cisapride, a non-selective 5-HT_4_ agonist, was the first effective prokinetic to be used as an add-on therapy in patients complaining of nocturnal heartburn (143). However, due to the report of a few cases of QT prolongation and fatal arrhythmias, it was withdrawn in the early 2000s. Other non- selective 5-HT_4_ agonists have also been evaluated. Mosapride also acts on 5-HT_3_ receptors through its metabolites, stimulating esophageal clearance and gastric emptying rate. Despite initial doubtful results (118), it seems to be effective in controlling rGERD symptoms when used as an add-on therapy (144), but its efficacy in true refractory patients still has to be proved. On the contrary, Revexepride, another 5-HT_4_ agonist, failed to achieve clinical benefits in r-GERD (145). Finally, Tegaserod, acting on both 5-HT_4_ and 5-HT_2B_ receptors, has been shown to be effective in decreasing reflux events and TLESR, but it has never been tested within the setting of rGERD. In more recent times, Prucalopride, a highly selective 5-HT_4_ agonist approved for the treatment of constipation, was also shown to be effective in reducing AET and in stimulating gastric emptying when used at high doses (e.g., 4 mg/die) in healthy volunteers (82). Despite its potential in stimulating secondary peristalsis in GERD patients, further studies are needed to assess its efficacy in achieving symptom control, particularly with regard to rGERD.

**Clinical tip: prokinetics may be used if delayed gastric emptying is associated**.

### Gamma Aminobutyric Acid-B Receptor Agonists and Metabotropic Glutamate Receptor-5 Antagonists

Gamma aminobutyric acid-B (GABA-B) receptor agonists and metabotropic glutamate receptor-5 (mGluR5) antagonists are drugs able to reduce the number of reflux events, irrespective of whether they are acid, weakly acid or alkaline. However, patients who could benefit the most are those with symptomatic weakly acid or bile reflux. Baclofen, a GABA-B receptor agonist, has been shown to decrease acid reflux events, esophageal acid exposure and reflux-related symptoms, both as a monotherapy for 2–4 weeks or as an add-on therapy to PPIs in patients with persistent symptoms (146). Its potential value as an add-on therapy was also confirmed in a recent meta-analysis (147). In order to overcome the short half-life and poor tolerability profile of Baclofen in terms of the central nervous system and abdominal side effects (i.e., dizziness, accommodation disorders, drowsiness, nausea, vomiting or diarrhea), new compounds with a greater binding ability for peripheral receptors have been developed. Among these, Lesogaberan, a GABA-B receptor agonist, was effective when used as an add-on therapy, with a dose-dependent mechanism (144). A phase IIb trial with more than 500 rGERD patients, showed clinical response only at higher doses (i.e., 240 mg/die), leading to a stop in further developments of Lesogaberan (148). Arbaclofen placarbil is the active R-isomer of Baclofen. Despite being initially found to be effective in controlling reflux events, a trial evaluating its efficacy when used in monotherapy in patients with symptomatic GERD found a lack of effects, decreasing enthusiasm regarding its development (149). Although a *post-hoc* analysis in patients with rGERD treated with Arbaclofen and PPI showed efficacy in controlling heartburn when compared to placebo (149), its development has also been interrupted. mGluR5 antagonists are drugs developed to act by targeting LES barrier function through inhibiting TLESR. Among these, Mavoglurant (150) and seemed to be promising in controlling GERD. In summary, despite several efforts in the development of new anti-reflux agents, their relatively scarce efficacy or the onset of side effects has led to a high rate of discontinuation (118). Thus, to date, in this class of drugs the only effective option for rGERD patients is that of adding Baclofen 5–10 mg three times a day, with close monitoring of neurological and/or abdominal side effects.

**Clinical tip: Among GABA-B receptor agonists baclofen is the only effective option for rGERD patients**.

### Pain Modulators: Antidepressants and Transient Receptor Potential Vanilloid Receptor-1 Antagonists

Persistence of reflux symptoms may be due also to esophageal hypersensitivity and hypervigilance for which it is hypothesized that mechanisms of central and peripheral sensitization are involved, also in correlation with response to stress. In these cases visceral analgesics [e.g., tricyclic antidepressants (TCAs), selective serotonin reuptake inhibitors (SSRIs), serotonin norepinephrine reuptake inhibitors (SNRIs) or trazodone] can be of help, particularly in avoiding the unnecessary use of high doses of acid inhibitory therapies in patients diagnosed as refractory to PPIs (151). These drugs can exert an analgesic effect through both peripheral and central nervous system pain modulation. To date, evidence for rGERD patients is however only indirect and comes from some placebo-controlled studies. For example, Citalopram 20 mg/day was found to be effective in patients with RH and Venlafaxine 75 mg/day obtained a good control of FH (152). Also Fluoxetine and Sertraline, compared with placebo and PPIs, improved symptoms in patients without pathological AET, in 6–8 weeks (149). It is to be highlighted that the administered doses did not interfere with mental functions because they were lower than those usually administered to obtain anxiolytic and/or antidepressive effects. Currently, there are no indications as to how long this therapy should last. Despite efficacy in reducing esophageal pain perception in healthy volunteers, TCAs (e.g., Imipramine, Nortriptyline) were not particularly able to treat functional esophageal symptoms (153). This is probably due to their action in delaying oro-cecal transit time (154). Transient receptor potential vanilloid receptor-1 (TRPV1) is an acid, heat and capsaicin sensitive receptor involved in the transduction of nociceptive reflux-induced stimuli. AZD1386 is aTRPV1 antagonist which seemed to be effective in increasing pain thresholds in healthy volunteers but did not lead to a significant symptomatic response in one study on rGERD patients (155). To date, no further studies evaluating this drug have been published. Summarizing, low doses of SSRIs and SNRIs are the only agents available and safe for patients complaining of reflux symptoms without evidence of pathological AET. An empirical approach suggested by Scarpellini et al. might be the association of an SSRI (Citalopram or Fluoxetine) at standard morning PPI doses, with follow-up evaluation of symptom improvement after 6 weeks (35). Additional studies are clearly needed, specifically those with a larger number of patients and a better stratification according to reflux parameters and psychiatric disorders.

**Clinical tip: if there is esophageal hypersensitivity and hypervigilance, SSRI and SRNI could be used at low doses**.

### Gastric-Retained Bile Acid Sequestrants

Gastric-retained bile acid sequestrants are an old-new class of drugs binding bile acids in the stomach. They represent an extended-release formulation of the bile acid sequestrant Colesevelam. Preliminary studies have shown potential benefits in rGERD patients. A recent clinical trial of a new compound (IW-3718) also proved to be effective in symptom control when the drug was used as an add-on to a standard PPI dose (98). The short-term use of these agents, when they are available on the market, could thus be a reasonable approach in patients with rGERD (156).

### Alternative and Complementary Therapies: Psychological Factors

Alternative therapies including psychotherapies, are becoming increasingly popular among patients with scarce response to PPIs doses or with reflux-like symptoms unrelated to pathological AET (157). Psychological factors can make esophageal symptoms worse and psychiatric comorbidities are often associated with persistent symptoms (158) and poor PPI response. In addition to the use of antidepressants (e.g., SSRI) at full dose (159), many other possible approaches have been suggested in recent years, despite the limited evidence associated with such methods. Among these, cognitive- behavioral therapy involving diaphragmatic breathing (160) and esophageal-directed hypnotherapy (161) could be interesting approaches in selected patients, targeting specific pathophysiological mechanisms such as supra-gastric belching (160) and functional pain, respectively. Hypnotherapy may also play a role in influencing gastric acid secretion and gastric emptying time, probably by modulating esophageal hypervigilance. Biofeedback therapy, a technique which employs visual and audio signals or direct verbal feedback to gain a greater awareness of bodily functions, could be interesting (162). Finally, also acupuncture is reported to improve GERD symptoms, although the exact mechanisms are not known (163). However, studies evaluating the clinical efficacy of these approaches in patients with rGERD are scarce.

## Endoscopic and Surgical Management

In the case of failure of medical therapies, invasive antireflux options should be considered (87, 93, 164). The most widely performed invasive antireflux option remains laparoscopic antireflux surgery (LARS) (14, 164–166), even if other, less invasive, methods have been suggested in the last few decades (167, 168). These include endoscopic transoral incisionless fundoplication (TIF), magnetic sphincter augmentation (LINX) or radiofrequency therapy (Stretta). It is mandatory to choose the most suitable option following a discussion with the patient regarding the relative risks and benefits (e.g., long-term efficacy, invasiveness, etc.).

### Laparoscopic Antireflux Surgery

The targets of surgical fundoplication are to reposition the LES in the abdomen, closing the hiatal opening, and creating a 1-way flap valve (169). LARS has a success rate ranging from 67 to 95%, greatly depending on adequate patient selection and preoperative evaluation, and on the practitioner's expertise (170–172). Post fundoplication symptomatic recurrence can be caused by an incorrect indication and/or an incomplete preoperative evaluation, or by an inadequate surgical technique (173). Older age, female sex, and presence of comorbidities are reported as risk factors for symptom recurrence (174). In a recent randomized controlled trial (RCT), including patients with GERD refractory to a course of Omeprazole 20 mg twice daily for 2 weeks, the success of the treatment with laparoscopic Nissen fundoplication at 1 year was significantly superior to that obtained with active medical treatment (Omeprazole plus Baclofen, with Desipramine added depending on symptoms), or control medical treatment (Omeprazole plus placebo) (44). LARS can be total or partial. Nissen is a total (360°) fundoplication after crural closure. The two most common partial LARS include a 270° posterior fundoplication (Toupet) and a 180° anterior fundoplication (Dor) (175). A RCT showed a similar effect of partial and total LARS for controlling GERD 3 years after surgery (176). Up to 30% of patients will develop structural complications, often related to surgical positioning or construction of the wrap (177). The EGJ is a complex anatomical area subjected to a multitude of mechanical stresses, related to the gastroesophageal pressure gradient and its possibility of moving axially. Thus, the fundoplication may weaken over time, resulting in wrap disruption, herniation or slippage (178–180). Approximately 5–10% of patients undergoing LARS can receive a second procedure to control their symptoms over time (181). Furthermore, an excessively tight fundoplication could provoke dysphagia (3, 139, 182). According to Håkanson et al., partial LARS is associated with lower rates of post-procedure dysphagia at 2 years in comparison with total LARS (176), particularly regarding anterior fundoplication (183). The partial LARS may be preferred to reduce post fundoplication dysphagia within the setting of an impaired esophageal peristaltic reserve at baseline (175). HREM, performed at the time of diagnosis to rule out esophageal motility disorders is also useful before surgery to assess esophageal contractile vigor and reserve, addressing the choice of the most suitable type of operation (87). Indeed, gastrointestinal symptoms following LARS are not uncommon, including dysphagia, gas-bloating syndrome, chest pain, and diarrhea (177). Consequently, LARS should be recommended with caution, as it can provoke severe adverse effects and the intended effect may be only temporary, because up to 60% of patients will use antireflux medical therapy in the following decade (184). Moreover, among patients who underwent primary LARS, 17.7% experienced recurrent GERD-like symptoms, which required long-term medication or secondary antireflux surgery (174).

### Alternative Invasive Procedures

Several attempts have been made to develop minimally invasive, mainly endoscopic, procedures to improve the antireflux barrier (185). In the last two decades, several antireflux approaches have become available. All these procedures act on different mechanisms involved in GERD pathophysiology, in particular EGJ reconstructive therapies and/or LES augmenting therapies.

#### Endoscopic Transoral Incisionless Fundoplication

TIF is an endoscopic procedure which aims to repair hiatal hernia and restore the LES physical barrier by creating a mechanical valve through a partial fundoplication (186, 187). TIF 2.0, a current technique iteration, is anatomically and physiologically similar to LARS. During the procedure, the gastric fundus is folded up and around the distal esophagus and anchored with polypropylene fasteners. TIF 2.0 has been shown to be a safe and effective treatment only for GERD patients with a hiatal hernia of <2 cm and a Hill grade of <3. In fact, patients with a hernia larger than 2 cm or Hill grade 3–4 should be candidates for LARS or laparoscopic hernia repair with concomitant TIF (188). In a cohort study of 49 patients, followed up to 10 years, most patients (92%) had stopped or reduced the use of PPI therapy after TIF (189). A recent meta-analysis was conducted using data only from RCTs that assessed the TIF 2.0 procedure compared with sham or PPI therapy. Its aim was to determine the efficacy and long-term outcomes associated with TIF 2.0 in patients with rGERD using optimized PPI therapy. Results from this meta-analysis demonstrated that the TIF 2.0 procedure at 3 years produced significant changes in esophageal pH, decreased PPI utilization, and improved quality of life (190). It is to be highlighted that among the studies included in the meta- analysis only one applies the definition of rGERD (187, 191, 192). Furthermore, in another meta- analysis, TIF was not superior to LARS regarding improvement of esophagitis and increase in LES pressure (186, 193). The serious adverse event rate, including gastrointestinal perforation and bleeding, ranges from 2 to 2.5% (194, 195).

#### Medigus Ultrasonic Surgical Endostapler Procedure

MUSE is a novel endoscopic device that closely mimics surgical anterior fundoplication through transoral stapling. It appears to be safe and technically easy (196). In a 6-month prospective trial, MUSE was reported to have improved symptom control and reduced PPI use in patients with ≥2 years of documented GERD symptoms and ≥6 months of continuous PPI therapy (197). These results were confirmed in a long-term trial involving 37 patients treated with MUSE; they were similar or better in efficacy and safety than TIF 2.0 and Stretta procedures (198). However, larger studies with control groups are needed to determine the real long-term efficacy of MUSE for GERD and rGERD patients.

#### Endoscopic Mucosal Resection Techniques

Endoscopic mucosal resection therapies differ in technical aspects, but substantially consist in an endoscopic ablation of cardial mucosa, causing LES tightening due to the scarring process, and includes anti-reflux mucosectomy (ARMS) (199), resection and plication (RAP) (200), anti-reflux mucosal ablation (ARMA) (201), antireflux ablation therapy (ARAT) (202), mucosal ablation and suturing of the EGJ (MASE) (203). According to the various authors, these procedures have been performed in PPI-refractory GERD patients, but the current definition of rGERD has only been used in ARMA and ARAT studies. Stenosis and/or dysphagia during post-procedure follow-up have been reported in some patients. The post-procedural efficacy has been variably assessed considering a decrease in AET and/or DeMeester (DM) score, GERD-Health Related Quality of Life Questionnaire, GerdQ and Frequency Scale for the Symptoms of GERD, and PPI discontinuation. Even if most studies report significant improvement in the evaluated parameters, they are flawed by many limitations such as lack of multicenter RCTs, small patient samples and short-term evaluations (199–203).

#### Endoscopic Full-Thickness Plication (GERDx)

Endoscopic full-thickness plication with the GERDx device was performed in 40 patients with GERD not responding to PPI use for ≥6 months. The most common adverse events were sore throat (20%) and chest pain (17.5%). Ten percentage of patients developed serious postoperative adverse events and about 17.5% underwent LARS before the 3-month follow-up. In the thirty patients available at the 3-month follow-up, an improvement of DM score, reflux related symptoms and gastrointestinal quality of life index were observed. However, three patients needed PPI treatment daily and eight on demand at the 3-month follow-up (204). In conclusion, the GERDx device does not seem to display satisfactory efficacy and safety for rGERD patients.

#### Magnetic Sphincter Augmentation (LINX)

Magnetic Sphincter Augmentation with the LINX device is a reversible procedure approved by the FDA for treatment of rGERD that does not alter the esophageal and gastric anatomy (177, 205). The device, laparoscopically placed around the LES, is aimed at creating an effective antireflux barrier, thus allowing bolus transit into the stomach and enabling belching and vomiting (206). Recent research by Rogers et al. showed that LINX is indicated in patients with a high number of reflux episodes (specifically > 80) detected on MII-pH monitoring, and reducing the number of reflux events to physiological levels provides an effective improvement in symptom outcome and treatment satisfaction (207). However, the most common side effect of LINX is dysphagia, whereas the most feared complications are device migration and esophageal erosion (~0.15%) (208). A systematic review comparing LINX to LARS reported a similar efficacy in controlling GERD symptoms and esophageal pH, with a larger reduction in gas bloating and improvement in belching in patients receiving the LINX procedure (209). Currently, the device is not licensed for use in severe erosive esophageal disease or large hiatal hernias (210). LINX therefore represents an appealing alternative to LARS, but a RCT between these two interventions for the treatment of GERD is necessary in order to directly evaluate the real efficacy of LINX (209).

#### Radiofrequency Therapy (Stretta)

Stretta procedure delivers endoscopic radiofrequency energy to the mucosa around the LES, improving the barrier function of the EGJ through scar tissue formation (211). In 2013 the Society of American Gastrointestinal and Endoscopic Surgeons recommended Stretta with a high level of evidence for rGERD (212). However, conflicting results regarding the efficacy of Stretta for the management of GERD symptoms, decrease in PPI use and reduction of AET were all reported (213–215). A revision of 140 Stretta-related papers performed by Das et al. suggest that further studies are required to evaluate the long-term efficacy of Stretta compared to the current best medical and surgical treatments for patients with GERD (216).

#### Esophageal Neurostimulation

An electrical neuromodulator (EndoStim), implanted into the abdominal wall by laparoscopy with a pair of electrodes placed on the LES, has been approved in Europe and South America. In an uncontrolled before-after study the device has proved to reduce symptom scores and AET in GERD patients with incomplete response to daily use of PPI for ≥12 months. The serious adverse events reported related to a device or procedure were bowel perforation and lead to erosion only in 2 out of 43 patients (217). Overall, the efficacy of neurostimulation in rGERD is still uncertain (218).

**Clinical tip: if there is failure of medical therapies, LARS remains the gold standard among invasive antireflux options**.

## Discussion and Conclusions

rGERD is a complex condition, and undoubtedly its non-univocal definition makes it difficult to compare different studies and draw appropriate and definite conclusions. Solving this not exclusively semantic problem could therefore be an effective starting point for planning consistent and effective clinical trials. In rGERD the persistence of symptoms is often associated with pathophysiological mechanisms involving also non-gastrointestinal systems and is still not fully understood. Moreover, symptoms of rGERD do not depend only on the presence of actual reflux but on other factors that are often difficult to distinguish, i.e., RH and FH. The first line strategy should always include a thorough reassessment of dietary and lifestyle factors, reiterating correct PPI timing and dose modalities. Additional pharmacological therapy should target the potential underlying pathophysiology according to the results of instrumental diagnostic tests (e.g., EGD, HREM, MII-pH monitoring and gastric emptying tests). When considering further therapy for rGERD, however, physicians should keep well in mind that evidence is often weak or indirect, coming from small studies that are often not RCTs. PPI therapy can be widely modulated according to the results of MII-pH monitoring, with a double dose (if not already suggested) if there is ineffective acid suppression or a split dose when there is NAB. Shifting to another PPI molecule, especially a cytochrome-independent molecule, can be suggested especially in populations where the prevalence of RMs is higher. A rescue trial with high-dose PPIs (i.e., q.i.d.) is a less valid option due to its weak evidence of efficacy, the need for high-grade compliance, the higher costs and the possible higher risk of side effects. PPI MR, where available, can be a valid alternative in patients with low compliance, as they require only a single administration per day. It is worth recalling that PPI therapy should always be titrated to the lowest minimal effective dose after a 4–8 week full-dose period, in order to prevent potential long-term side effects. However, their efficacy is not usually long lasting due to tachyphylaxis phenomena. They can thus be used for up to 4-week periods, possibly restarting them after a 4 week washout. Alginates and mucosal protective agents can be a valid alternative when used as an add-on therapy to PPIs within this clinical context, given the absence of significant side effects. Sucralfate, alginates, or mucosal protective agents, together with GABA-B receptor agonists, can also be an effective option if there is persistence of non-acid or weakly acid reflux. Baclofen is the only effective GABA-B receptor agonist available on the market, but due to its many potential side effects, it should be used in very carefully selected cases. Adding a prokinetic with close monitoring for side effects could be an option when there is concomitant delayed gastric emptying or gastroparesis. However, it should be mentioned that these agents are effective only with reflux symptoms associated with altered motor gastric function and their efficacy has not been thoroughly evaluated.

**Figure 2:**
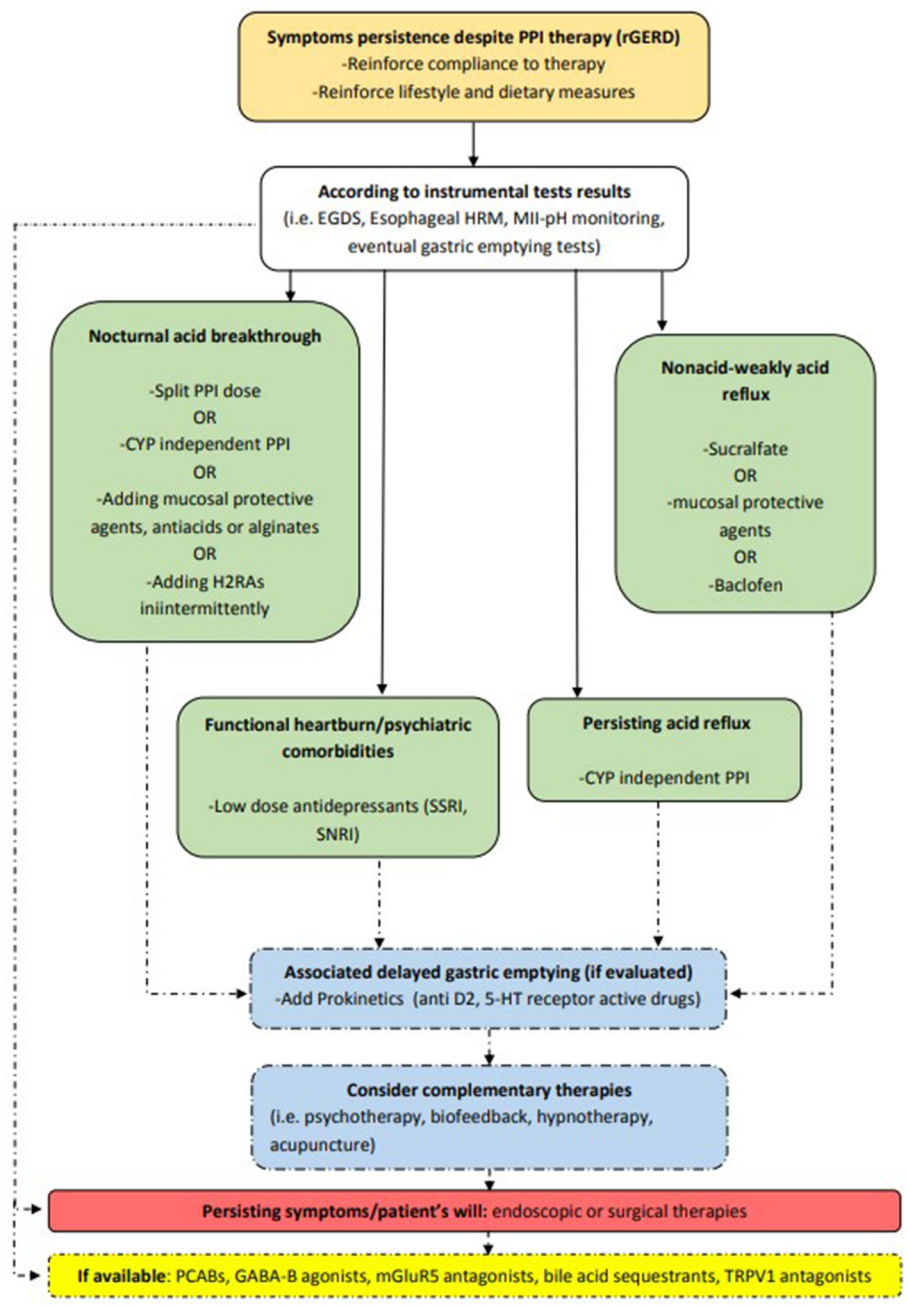
Different therapeutic options in rGERD management according to pathophysiology. CYP, cytochrome P450; D2, dopamine 2; EGDS, esophagogastroduodenoscopy; EoE, eosinophilic esophagitis; GABA-B, gamma aminobutyric acid-B; H2RAs, histamine receptor 2 antagonists; HRM, high resolution manometry; mGluR5, metabotropic glutamate receptor-5; MII-pH, multichannel intraluminal impedance-pH; PCABs, potassium-competitive acid blockers; PPI, proton pump inhibitor; rGERD, refractory gastroesophageal reflux disease; SNRI, serotonin and norepinephrine reuptake inhibitor; SSRI, selective serotonin reuptake inhibitor; TRPV1, transient receptor potential vanilloid receptor-1; 5-HT, 5-hydroxytryptamine.

If RH or FH are detected, or the patient suffers from psychiatric comorbidities, pain modulation with antidepressant drugs should be the choice to control symptoms, either singly or in combination with standard PPI doses. Low dose SSRIs seem to be safer than the older tricyclic antidepressants, although a thorough evaluation in rGERD patients is still lacking. Finally, many new agents targeting potential pathogenic mechanisms are currently being developed or are awaiting approval in Western countries and are soon expected to deal with the complexity of rGERD. Among these, PCABs seem to be the most promising agents, but further studies are needed to evaluate their potential long-term side effects. Complementary therapies have not been thoroughly evaluated to date, but some of them (e.g., cognitive-behavioral therapy and biofeedback) are interesting in patients where psychological factors can play an important role in eliciting or worsening symptoms. When medical management fails, invasive antireflux options should be evaluated after having carefully explained their risks and benefits to the patient. An objective confirmation of rGERD is mandatory because surgical and endoscopic antireflux interventions are invasive procedures and outcomes largely depend on appropriate patient selection. The gold standard option remains LARS. This remains true, even if other less invasive interventions, which have not yet demonstrated long-term safety and efficacy, have been suggested in recent years. Figure 2 provides an overview of possible treatments for rGERD based on its pathophysiology.

## Author Contributions

FR, FB, NB, and MB: conceptualization and methodology. FR, FB, MC, and MB: writing—original draft preparation. CL, AP, GB, ES, NB, and FZ: review and editing of final manuscript. SM: supervision. All authors have read and agreed to the published version of the manuscript.

## Conflict of Interest

FZ was employed by company CHU de Bordeaux. The remaining authors declare that the research was conducted in the absence of any commercial or financial relationships that could be construed as a potential conflict of interest.

## Publisher's Note

All claims expressed in this article are solely those of the authors and do not necessarily represent those of their affiliated organizations, or those of the publisher, the editors and the reviewers. Any product that may be evaluated in this article, or claim that may be made by its manufacturer, is not guaranteed or endorsed by the publisher.
